# Editorial: Computational multimodal sensing and perception for robotic systems

**DOI:** 10.3389/frobt.2026.1967986

**Published:** 2026-10-26

**Authors:** Shihan Lu, Yawen Liu, Youcan Yan, Jianzhe Gu, Jingxian Wang, Alex Wong, Heather Culbertson

**Affiliations:** 1 Center for Robotics and Biosystems, Northwestern University, Evanston, IL, United States; 2 Electrical and Computer Engineering Department, Carnegie Mellon University, Pittsburgh, PA, United States; 3 UMR5506 Laboratoire d’Informatique, de robotique et de microélectronique de Montpellier (LIRMM), Montpellier, France; 4 School of Computer Science, Carnegie Mellon University, Pittsburgh, PA, United States; 5 Department of Computer Science, National University of Singapore, Singapore, Singapore; 6 Department of Computer Science, Yale University, New Haven, CT, United States; 7 Department of Computer Science, University of Southern California, Los Angeles, CA, United States

**Keywords:** cross-modal alignment, learning-based control, multimodal sensing, representation learning, robotic perception, sensor development, sensor fusion

## 1 Introduction

Each sensor captures its own slice of the physical world. For robots working in complex and dynamic environments, robust perception requires integrating complementary information from multiple sensory modalities. Vision may provide global context, while tactile, auditory, event-based, and other emerging sensing technologies can reveal more interaction details that are difficult to obtain by vision alone. Together, these modalities can support more robust and adaptive robotic behaviors.

Despite substantial progress, integrating heterogeneous sensory signals remains challenging because different modalities vary in their spatial and temporal characteristics, representations, and levels of uncertainty. Existing approaches often rely on task-specific sensor configurations and carefully designed fusion pipelines, limiting their generalizability across robots and applications. Addressing these limitations requires advances in sensor design, signal processing, cross-modal representation, learning architectures, and distributed perception frameworks.

This Research Topic highlights recent developments in multimodal sensing and perception for robotic systems and related applications, such as virtual reality, across *Frontiers in Robotics and AI* and *Frontiers in Virtual Reality*. It features contributions on multimodal sensor design, cross-modal signal alignment and fusion, multimodal representation learning, and distributed or coordinated sensing systems, along with their applications in robot manipulation, navigation, and human–robot interaction. By bridging advances in multimodal sensing technologies with computational perception, this collection aims to foster the development of more capable, adaptable, and intelligent robotic systems.

## 2 Summary of the articles

The five articles collected in this Research Topic address complementary layers of multimodal perception, spanning sensing integration, multimodal representation, sensor calibration, embodied reasoning, and human–agent interaction.

Full-stack research integrating sensor development, signal representation, and learning-based control is essential for translating advances in multimodal perception into practical robotic capabilities. Addressing the challenges of extracting and integrating high-dimensional tactile information, Ning et al. introduce TouchWGNN, a multimodal framework for dexterous in-hand manipulation that represents tactile measurements as spatio-temporal graphs. The authors first develop a low-cost array comprising 113 taxels distributed across a five-fingered robotic hand. A graph-based spatial encoder captures contact geometry, while a temporal module models how contacts evolve over time. By fusing features from tactile measures with vision and proprioception into a policy based on reinforcement learning, the system improves object-state estimation and performance in cube reorientation and Baoding ball swapping, particularly when visual observations are degraded.

The sim-to-real gap in high-dimensional sensing modalities remains another major obstacle to scalable policy learning and deployment. Lin et al. address this challenge for magnetic tactile sensing, presenting a unified framework for simulating and representing tactile signals in precise robotic assembly. Their approach uses the Material Point Method to model elastomer deformation as physically grounded point clouds, while Point-PAMAE compresses this high-dimensional data into compact latent representations that preserve contact-relevant geometry. A latent-space Real-to-Sim mapping then aligns real magnetic sensor readings with simulated deformation features, bridging the two domains. The results demonstrate high-resolution simulation with modest memory overhead and robust cross-domain alignment, providing a foundation for tactile-driven sim-to-real learning in precision assembly tasks.

Cross-modal alignment poses its own challenges, as sensing modalities differ significantly in their physical principles, data structures, resolutions, and susceptibility to noise. Chen and Sun propose a targetless method for maintaining LiDAR-camera extrinsic calibration during long-term robotic operation. Rather than relying on artificial calibration targets or discrete semantic labels, the method aligns continuous semantic probability distributions between LiDAR and camera data. Anchor-fixed sampling stabilizes the optimization objective, direction-aware weighting improves yaw observability, and a globally balanced Jensen–Shannon divergence mitigates semantic class imbalance and noise. Evaluations on the KITTI Odometry dataset show reliable convergence even from substantial initial perturbations, highlighting the method’s potential for robust field recalibration.

Beyond sensory fusion, robust robotic perception also requires representations that support interpretable and verifiable reasoning. Dai presents an opinion article on structure-aware visual reasoning as a complement to end-to-end vision–language models in robotics and safety-critical applications. While acknowledging the strong semantic capabilities of modern models, the article emphasizes persistent risks, including hallucination, incorrect attribute binding, relational inconsistency, and limited constraint enforcement. It argues that model outputs should be treated as uncertain hypotheses and organized into explicit, inspectable representations of entities, attributes, relations, temporal states, constraints, and uncertainty, enabling consistency checking and verification.

Multimodal cues also play a central role in shaping human perception and social interaction. Richard et al. investigate how visual, auditory, and tactile feedback influence the perception of social touch initiated by a virtual agent. Across three experiments, the authors examine touch recognition, believability, and agency attribution under a range of unimodal and multimodal conditions. Visual feedback provides the strongest guidance for gesture recognition, while tactile and auditory cues help resolve ambiguity and strengthen the sensation of being touched. The findings further reveal that animation quality and interpersonal distance shape whether the virtual agent is perceived as the intentional source of touch, underscoring the importance of both multisensory congruence and plausible interaction context in human-agent interaction.

These contributions illustrate that advancing multimodal robotic perception requires progress across several connected dimensions. Collectively, they show that effective multimodal perception depends on representations and mechanisms that capture complementary information, align heterogeneous signals and domains, and support robust sensing, reasoning, and interaction across diverse tasks and environments.

## 3 Outlook

Multimodal robotic perception should not be limited to reproducing human sensing mechanisms. While biological perception offers valuable inspiration, robots can exploit sensing principles that reveal physical properties and interaction states that are inaccessible or imperceptible to humans. These may include internal material properties, non-contact field perturbations, and high-bandwidth contact mechanics. Co-designing such sensors with the computational representations used to interpret their outputs could enable robots to infer hidden states, detect subtle interactions, and operate reliably when conventional vision, touch, or audio are insufficient.

At the same time, progress in multimodal perception depends not simply on increasing the number of sensors, but on understanding the distinct contribution of each modality. Different sensory channels vary in spatial coverage, temporal resolution, physical meaning, and sensitivity to environmental disturbances. A modality that contributes little under nominal conditions may become essential during occlusion, sensor degradation, rapid contact transitions, or distribution shifts. Future systems should learn when and how to draw on each modality, dynamically adjusting their reliance on sensory inputs according to task context, uncertainty, and signal quality. This calls for more systematic evaluation of modality complementarity, redundancy, and failure modes, instead of treating multimodal fusion as the straightforward aggregation of all available signals.

Another important direction lies in integrating emerging sensing modalities into large-scale foundation models and general-purpose perceptual backbones. Most existing multimodal models are built primarily around vision and language, while robot-relevant signals like touch, sound, and proprioception are often handled through task-specific modules or narrow fine-tuning. Developing scalable methods for tokenization, alignment, pretraining, and adaptation could help unify these heterogeneous signals within a shared latent space. Such foundation models could facilitate transfer across sensors, robotic embodiments, tasks, and environments, while reducing the need to design custom fusion architecture for each sensor configuration and application.

